# An electrocorticographic BCI using code-based VEP for control in video applications: a single-subject study

**DOI:** 10.3389/fnsys.2014.00139

**Published:** 2014-08-07

**Authors:** Christoph Kapeller, Kyousuke Kamada, Hiroshi Ogawa, Robert Prueckl, Josef Scharinger, Christoph Guger

**Affiliations:** ^1^Guger Technologies OGGraz, Austria; ^2^g.tec medical engineering GmbHSchiedlberg, Austria; ^3^Department of Computational Perception, Johannes Kepler UniversityLinz, Austria; ^4^Department of Neurosurgery, Asahikawa Medical UniversityAsahikawa, Japan

**Keywords:** electrocorticography, brain-computer-interface, code-based stimulation, VEP, augmented control

## Abstract

A brain-computer-interface (BCI) allows the user to control a device or software with brain activity. Many BCIs rely on visual stimuli with constant stimulation cycles that elicit steady-state visual evoked potentials (SSVEP) in the electroencephalogram (EEG). This EEG response can be generated with a LED or a computer screen flashing at a constant frequency, and similar EEG activity can be elicited with pseudo-random stimulation sequences on a screen (code-based BCI). Using electrocorticography (ECoG) instead of EEG promises higher spatial and temporal resolution and leads to more dominant evoked potentials due to visual stimulation. This work is focused on BCIs based on visual evoked potentials (VEP) and its capability as a continuous control interface for augmentation of video applications. One 35 year old female subject with implanted subdural grids participated in the study. The task was to select one out of four visual targets, while each was flickering with a code sequence. After a calibration run including 200 code sequences, a linear classifier was used during an evaluation run to identify the selected visual target based on the generated code-based VEPs over 20 trials. Multiple ECoG buffer lengths were tested and the subject reached a mean online classification accuracy of 99.21% for a window length of 3.15 s. Finally, the subject performed an unsupervised free run in combination with visual feedback of the current selection. Additionally, an algorithm was implemented that allowed to suppress false positive selections and this allowed the subject to start and stop the BCI at any time. The code-based BCI system attained very high online accuracy, which makes this approach very promising for control applications where a continuous control signal is needed.

## Introduction

People have long sought to extract users' intentions from brain signals to give impaired persons a communication channel or optimize interaction between users and their environments. Such a brain-computer-interface (BCI) allows the user to control a device or software with brain activity (Wolpaw et al., 2002). People can use a BCI to interact with their environments even if they have limited or no muscle control. Various data acquisition techniques like electroencephalography (EEG) (Wolpaw et al., 2002), electrocorticography (ECoG) (Leuthardt et al., 2004), functional magnetic resonance imaging (fMRI) (Weiskopf et al., 2004), and near infrared spectroscopy (NIRS) (Coyle et al., 2004) can be used as a BCI system.

The EEG is the most common brain imaging method in BCI research because it is inexpensive, portable, non-invasive, and has excellent temporal resolution (Mason et al., 2007). However, the EEG has only a limited spatial resolution, as each channel is influenced by the activation of millions of neurons, and the signal is smeared and filtered during passage through the scalp. ECoG signals recorded from the brain's surface are more robust against electromyographic (EMG) artifacts and provide a higher spatial and temporal resolution compared to EEG signals (Leuthardt et al., 2004). Several groups investigated the reliability of electrocorticographic signals for real-time applications, like 2D movement control based on motor imagery tasks (Schalk et al., 2008) or a P300 spelling device (Brunner et al., 2011).

Most BCIs rely on one of three kinds of brain signals: event related desynchronization (ERD) associated with motor-imagery, P300 and steady-state visual evoked potentials (SSVEP) (Wolpaw et al., 2002). While P300 based systems typically rely on discrete control, which is excellent for selecting commands to control a spelling device (Mason et al., 2007; Guger et al., 2009), the motor imagery and SSVEP based systems often give a continuous control signal that could be used to steer a wheelchair in different directions. The performance of a BCI relying on motor imagery has already been comprehensively investigated using EEG (Müller-Gerking et al., 1999; Guger et al., 2000, 2003; Krausz et al., 2003; Blankertz et al., 2010) and ECoG recordings (Leuthardt et al., 2004; Schalk et al., 2008; Miller et al., 2010). Recently, an ECoG BCI was presented that decoded attempted arm and hand movements for 3D cursor control using activation of the high-frequency band between 40 and 200 Hz, which were mapped to velocity control signals (Wang et al., 2013).

This work is focused on BCIs based on visual evoked potentials (VEP) derived from subdural ECoG signals over the visual cortex. Two methods are generally used to distinguish different visual targets: phase coding and frequency coding (Wang et al., 2008). In SSVEPs, the brain waves derived from the scalp contain sinusoidal signals with the same frequency as the visual stimuli. The so called Bremen BCI used a minimum energy based feature extraction applied on EEG data including SSVEPs and reached a mean ITR of 61.70 bits/min and a mean accuracy of 96.79% (Volosyak, 2011). Another SSVEP BCI study presented a grand average accuracy of 95.50% for EEG recordings from 53 subjects (Guger et al., 2012). An alternative type of stimulation that combines the phase and frequency coded VEPs is based on code sequences, producing code-based VEPs (c-VEP) (Bin et al., 2009). This type of BCI was initially proposed by Erich Sutter as a spelling device and tested with an ALS patient having epidural implants over 11 months (Sutter, 1992). The subject achieved a spelling rate of 10–12 words per minute. In a more recent paper, a multi-channel c-VEP BCI was tested with EEG signals (Bin et al., 2011). A spatial filter was computed from a canonical correlation analysis (CCA) and then applied on multiple EEG channels over the visual cortex. The authors developed a 32 target system with a stimulation sequence of 1.05 s. The resulting mean online accuracy was 85.00%, which led to an ITR of 108 bits/min. An even faster version of this c-VEP speller application led to an average accuracy of 96.00% and an ITR of 144 bit/min (Spüler et al., 2012). Although the code-based stimulation approach is not the same as a conventional steady state stimulus, BCIs based on c-VEP are often grouped together with SSVEP BCIs for convenience.

BCIs relying on VEPs could benefit from on-screen solutions, which do not need additional hardware like LEDs and can be embedded into a virtual environment. This was achieved inside a 2D or 3D gaming environments, as well as virtual reality scenarios (Lalor et al., 2005; Martinez et al., 2007; Faller et al., 2010). In a previous study we tested a c-VEP BCI for robotic control with EEG on 11 subjects showing a grand average online accuracy of 98.18% (Kapeller et al., 2013). Specifically, the control interface was embedded inside a video application showing the moving robot, while the user was steering the robot along a given path.

The main goal of this study is to investigate an intracranial c-VEP BCI designed as a continuous control interface for augmentation of video applications.

## Materials and methods

### Subject description

The subject, a 35 year old woman suffering from intractable epilepsy underwent subdural grid implantation prior to resective brain surgery, which was part of the clinical treatment. After full explanation of the experimental procedure and its possible risks, a written informed consent was obtained from the subject. This work was approved by the institutional review board of the Asahikawa Medical University (No. 693) before the study. She had corrected-to-normal vision and a Japanese Wechsler Memory Scale—Revised Edition (WMS-R) test showed normal memory function (average score 100, *SD* = 15). As the epileptic focus did not overlap with the visual cortex, there was no reason to reject the subject from the study.

**Figure 1:**
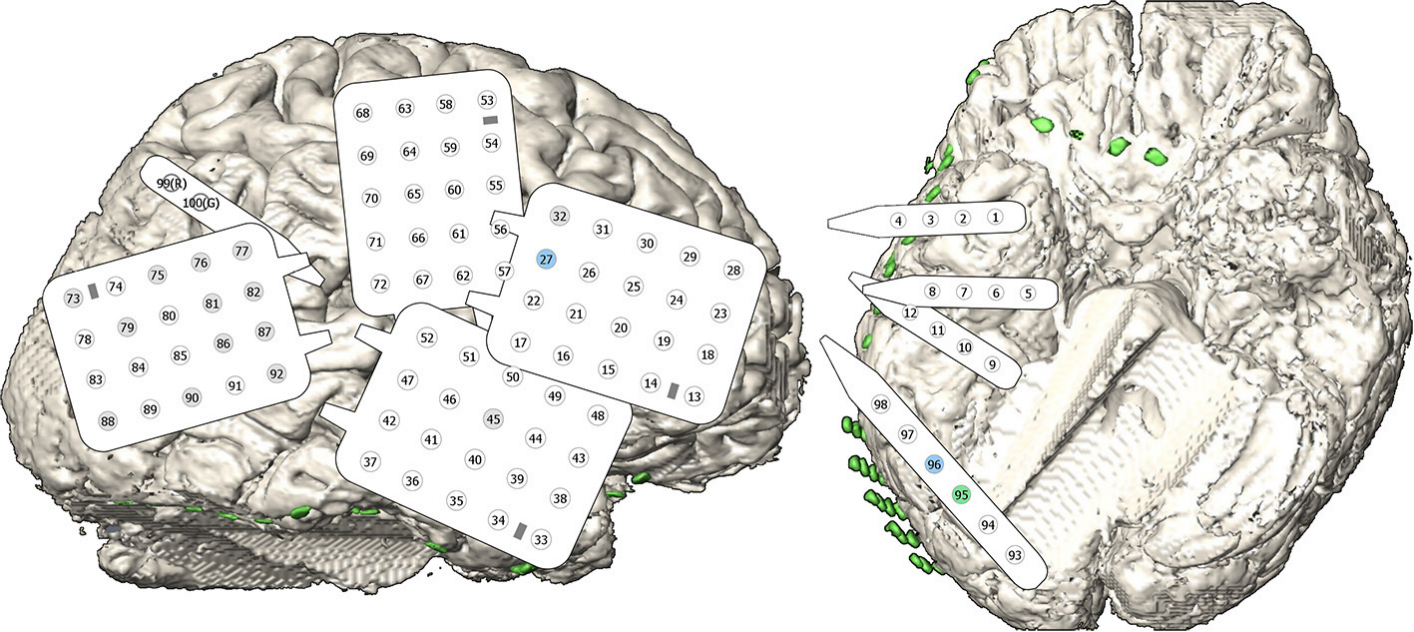
**Implanted electrodes**. The subject had 100 electrodes on **four** grids and **five** strips located implanted over the frontal, temporal, parietal, and occipital lobe of the right hemisphere. The schematic views of the grids and strips contain electrodes represented by a circle and their number in the montage. An additional strip on the frontal base region was not recorded and is not included into the electrode numbering. The gray highlighted electrodes represent very noisy channels which were recorded, but not considered for signal processing. The green/blue colored channels indicate high/medium contribution during feature extraction.

In order to localize epileptic foci, four grids and five strips containing 100 subdural electrodes were temporarily implanted widely across the right hemisphere. Figure 1 shows the grid alignment on the cortex and the numbering of the electrodes. The clinical electrodes were made of platinum and had an inter-electrode distance of 10 mm and a conductive area of about 7.1 mm^2^.

### Data acquisition

The electrodes were connected to a g.HIamp (g.tec medical engineering GmbH, Austria) biosignal data acquisition device, which digitized the data with 24 bit resolution and 256 Hz sampling frequency. The data were band-pass filtered between 1 and 30 Hz and notch filtered at 50 Hz to suppress power line interference. This pass band was designed to maximize the signal-to-noise ratio (SNR) of the recorded VEPs for a maximum stimulation frequency of 30 Hz using a monitor with a refresh rate of 60 Hz. The grounding strip covering the superior parietal lobule contained the electrodes number 99 and 100, which were used as reference (REF) and ground (GND) electrode, respectively. Therefore, 98 signal channels were recorded during the experiment.

The biosignal data acquisition device transferred the data via USB to the computer system, which was used for data acquisition, online processing, experimental paradigm control, data visualization and data storage.

### System configuration

The design of the BCI system is based on the work of Bin et al. (2011) and modified for continuous control input from the user. The stimulation unit, a 60 Hz LCD monitor, presents visual targets that each occupy 4.2 × 2.4 cm of the screen. These targets are embedded inside an OpenGL based video application and remotely connected with the BCI system, which controls the visual stimulation and the feedback to the user.

The stimulus definition is based on a pseudo-random binary code sequence, a so called m-sequence. These binary sequences are used for nonlinear signal analysis and also in multi input systems (Golomb et al., 1982). Therefore, black and white stimulation is applied using a 63 bit m-sequence, where 1 and 0 are represented by white and black, respectively. Due to the stimulation rate of 60 Hz, one stimulation sequence lasts 1.05 s. The BCI presented in this work contains four visual targets (C1, C2, C3, and C4), which can be selected by the user. This is a well-suited number of classes for a continuous control task, e.g., moving a robotic device or a virtual avatar forward, backward, left or right (Kapeller et al., 2013). As the autocorrelation function of the m-sequence is approximating the unit impulse function (Zierler, 1959), we can use the same modulation sequence for all visual targets. Only a phase shift of the sequence onset is necessary to distinguish between the resultant VEPs inside the synchronized processing unit of the BCI. The phase shift for each target is equally distributed along the m-sequence and then rounded to the next lower integer value. This leads to a phase shift in bits of ρ_1_ = 0, ρ_2_ = 15, ρ_3_ = 30, and ρ_4_ = 45 for class C1 to C4, respectively.

In order to synchronize the visual stimulation with the recorded ECoG signals, a trigger signal representing the sequence onset of C1 is sent via UDP connection to the BCI system. Once the stimulation is synchronized, the BCI is processing the resultant VEPs using a signal buffer that is updated each 51 samples (~200 ms). The processing system is running in the MATLAB/Simulink rapid prototyping environment.

### Screen overlay control interface (SOCI)

The visual targets are presented on the monitor through the SOCI module. The SOCI module is a C++ library based on OpenGL that is loadable at runtime and can be used by OpenGL based host applications to embed visual stimulation. The host applications could be virtual reality environments or simple real-world videos that are acquired from a camera. The SOCI works as a remote visual stimulator and provides a network interface that can be used to load visual targets, start or stop the flickering, and synchronize the stimulation with the signal processing. Since it is a multi-threaded application, the communication with the BCI model and the OpenGL drawing commands are handled within separate threads. The host graphics application has to initialize and control the SOCI. This utilizes an abstract interface class that provides public functions to embed the module.

The visual targets can be configured very flexibly using an XML description provided from a configuration file or an external application (Putz et al., 2011). Users can change the number, position, size, and content of the icons and modify other parameters.

### Experimental design

**Figure 2:**
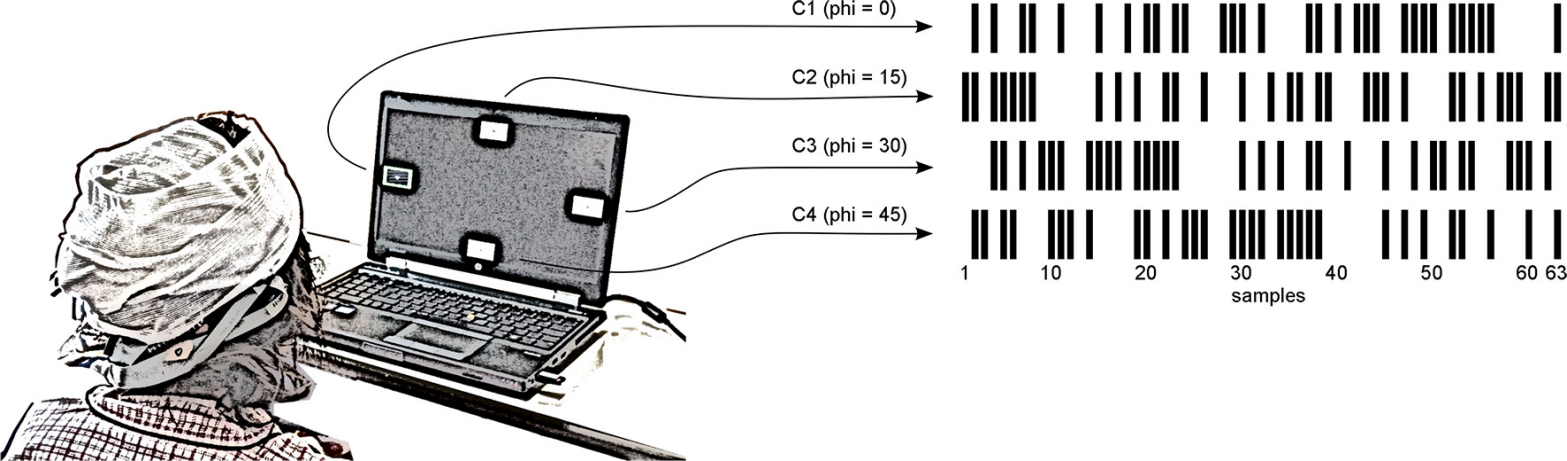
**Experimental design**. A notebook presented **four** visual targets to the subject, who sat around 80 cm in front of the monitor. The size of **one** target was 4.2 × 2.4 cm leading to a visual angle of 3.0 × 1.7°. Each visual target (C1, C2, C3, and C4) was flickering with a phase shifted version of the same m-sequence. The subject was asked to focus the cued target or to choose an arbitrary target if the cue was disabled. In unsupervised mode, the selected target was surrounded by a green border.

The subject was seated ~80 cm in front of the stimulation monitor visualizing the BCI targets during the experiment (see Figure 2), which was separated into three phases. Specifically, the subject participated in a calibration run to compute a classifier, an evaluation run to estimate the accuracy of the BCI and a free run to let the subject experience unsupervised interaction with the system in combination with online feedback of the selected target.

After a pre-run delay of 10 s (to avoid settling effects of the filters), the subject performed the calibration run. The subject's task was to focus on target C1 for 200 stimulation cycles, while all other targets were not visible. Since one cycle lasts 1.05 s, the entire calibration took less than 4 min.

In the evaluation run, all targets were displayed on the monitor and the online accuracy of the BCI system was tested across 20 trials. Specifically, the SOCI displayed four rectangles on the top, bottom, left, and right of the monitor (see Figure 2). During each trial, the subject focused on the target that was highlighted by a visual cue (green border around the target). Each trial consisted of a 3.0 s long resting phase with no visual stimulation, followed by a 7.0 s long stimulation period showing all targets flickering simultaneously. This trial definition was based on previous studies about BCIs using SSVEP or c-VEP (Guger et al., 2012; Kapeller et al., 2013). Since the stimulation period exceeded the longest buffer size and the signal buffer was updated each 200 ms, we decided to keep a trial duration that is not a multiple of the stimulation sequence. No feedback about the classification result was given to the subject at any time during the evaluation run.

Finally, the subject performed the free run to experience the interaction with the BCI by getting feedback about the selected target. Again, all four targets were flickering simultaneously with the m-sequence and corresponding phase shift. No cue was presented on the screen to indicate the current target, and all targets continued flickering throughout the free run. The subject could select any target at any time. Feedback reflecting the current selection was presented as a green border around the selected target. After the free run, the subject was asked about her impression of the system's accuracy.

### Offline analyses

The recorded data set from the calibration run was used for offline analyses. As we had to exclude bad channels from the offline analyses, all channels that did not pass visual inspection of the raw data were set to zero. After checking the signal quality, the 200 recorded sequences were epoched and DC corrected for each trial. Then the trials were averaged to a set of templates for each signal channel and phase shift. These templates were used for waveform detection of the c-VEP inside the ECoG signals. Therefore, a CCA was used to maximize the correlation coefficient of all templates and the raw data.

Compared to ordinary correlation, the CCA is independent from the used coordinate system. It provides the maximum correlation of the variables, which is also called the canonical correlation (Borga, 2001). The correlation coefficient ρ is maximized, with respect to the normalized base vectors for canonical correlation (*ŵ_x_* and *ŵ_y_*):

$$\underset{{}_{{\hat{w}}_{x}{\hat{w}}_{y}}}{\max}\rho =\underset{{\hat{w}}_{x}{\hat{w}}_{y}}{\max}\left(\frac{E[{\hat{w}}_{x}^{T}XY^{T}{\hat{w}}_{y}]}{E[{\hat{w}}_{x}^{T}XX^{T}{\hat{w}}_{x}]E[{\hat{w}}_{y}^{T}YY^{T}{\hat{w}}_{y}]}\right)$$

*X* and *Y* are the analyzed multidimensional variables. In our case, *X* contained the raw ECoG data of 200 sequences and *Y* consisted of 200 concatenated templates for each channel.

The calculation of the maximum correlation is based on the eigenvalue equations below, where C is the covariance matrix of the multidimensional variables. In these equations, multiple non-zero eigenvalues are possible. As the eigenvalues represent the squared correlation coefficients, the highest eigenvalue leads to the canonical correlation. The corresponding eigenvectors are the base vectors from:

$$\begin{array}{l} \;C_{xx}^{-1}C_{xy}C_{yy}^{-1}C_{yx}{\hat{w}}_{x}=\rho^{2}{\hat{w}}_{x}\\ C_{yy}^{-1}C_{yx}C_{xx}^{-1}C_{xy}{\hat{w}}_{y}=\rho^{2}{\hat{w}}_{y}. \end{array}$$

**Figure 3:**
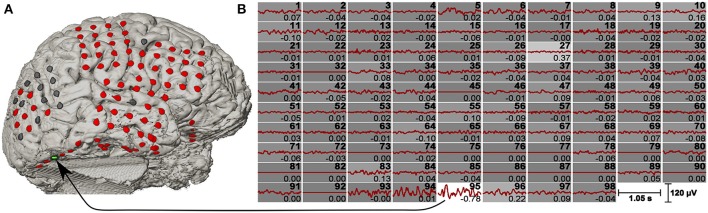
**c-VEP templates and spatial filter. (A)** Co-registered brain model of a pre-surgical T1-weighted MRI and a post-implantation CT scan. The channels are highlighted in different colors (bad channels are gray, normal channels are red, and the maximum weighted channel of the CCA is green). **(B)** Signals of all 98 channels were averaged over 200 stimulation sequences. Channel numbers are shown on the top right corner of each plot, while the spatial filter coefficients are shown on the bottom right corner. Beside the numeric value, the spatial filter coefficients are also represented by the background intensity of each channel (the highest weight is plotted on white background). Bad channels were set to zero.

The resultant vectors *ŵ_x_* and *ŵ_y_* were interpreted as spatial filters, where *ŵ_x_* was applied on the raw ECoG signals and *ŵ_y_* was applied on the template data. Figure 3 shows the averaged c-VEP templates for each channel together with the weight for the individual channel.

The spatial filtered calibration data was then used for computing a linear classifier using a multi-class linear discriminant analysis (m-LDA) (Duda et al., 2000). The features were extracted by computing the correlation coefficients of the spatially filtered templates and the spatially filtered raw data. In order to extract features for each class, the data set of the calibration run was epoched four times. Before each epoching step, the signals were shifted with respect to the trigger channel and the phase shift of the current class. Hence, the correlation coefficients were maximized for the corresponding phase shift.

A zero class provided an idle state that occurred when no target was selected by the user. Based on the classification scores only, it is not possible to determine whether the user has selected any target. This entails rejecting any classification result for which the residual error probability is larger than a predefined limit. A Softmax function is used, which transforms the output of the discrimination function into a value *p* between 0 and 1:

$$p_{i}=\frac{e^{q_{i/\tau }}}{\sum_{b\;=\;1}^{N}e^{q_{b/\tau }}}$$

Where *q_i_* is the distance to class *i* and τ is called the temperature, which is used to adjust the gap between the resultant probabilities (Sutton and Barto, 1998). *N* is the total number of possible classes.

### Setup for the online experiment

During the online experiments a Simulink model was processing incoming data with an update rate of 256 Hz driven by the data acquisition device. After the preprocessing step described in Section Data Acquisition, the recorded ECoG signals were buffered and DC corrected. The size of the signal buffer was set to a multiple of the template length and was updated every 51 processing steps. In order to extract characteristic features for each individual class, this buffer was then compared with the templates for each phase shift. Therefore, two processing steps were required. First, the spatial filter from the offline analysis was applied to the signal buffer and the templates and led to a combined channel with maximized correlation with respect to the spatially filtered templates. Second, the spatially filtered signal was correlated with each spatially filtered template leading to one feature channel for each class.

Before starting the free run, the data from the evaluation run was used to investigate the system's accuracy and latency. Different settings were tested to provide the best configuration for the free run.

As the length of the ECoG signal buffer could heavily influence the performance of the BCI, we tested the system accuracy for multiple buffer lengths. The signal buffer has to be a multiple of the m-sequence length, where the minimum length is the duration of one m-sequence cycle. We explored buffer lengths of 1.05, 2.10, and 3.15 s. Longer buffer lengths were not considered in this work, since this would not proper to our previously proposed BCI applications.

In order to stabilize the features, a 1.0 s moving window filter was used before applying the linear classifier. If the classification result was below a 97–100% confidence interval, the output was assigned to the zero class.

The mean online accuracy of the BCI was determined by averaging all classifier outputs at the last 2 s of each trial. This gives a measure about the performance of the BCI, when the feature values reached their maximum with respect to the selected target.

## Results

### Classification accuracy

**Figure 4:**
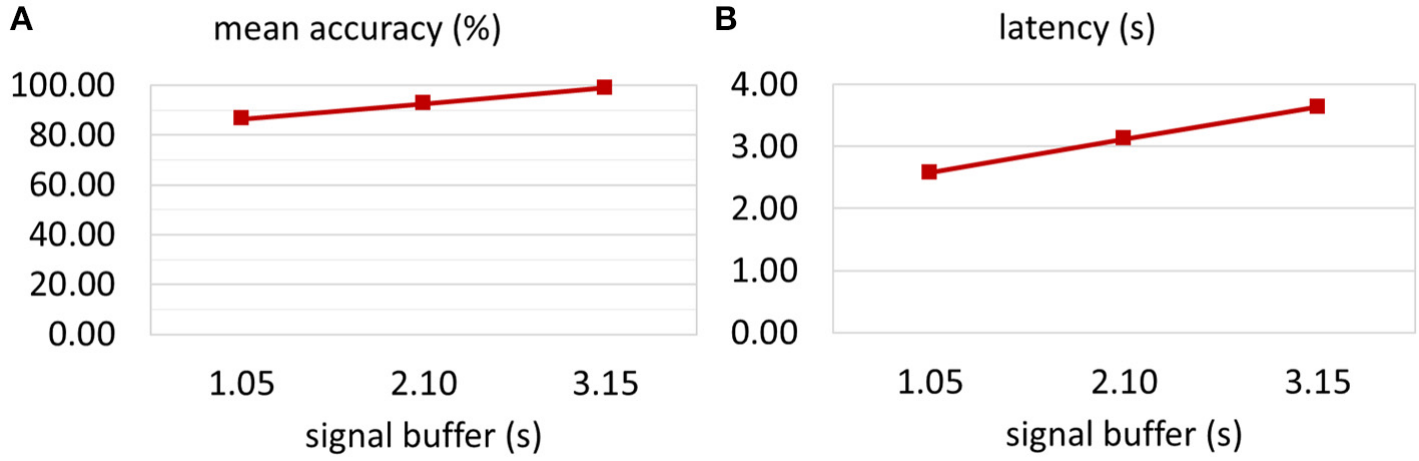
**Optimization of the signal buffer**. The classification accuracy for **three** different signal buffer sizes was tested. **(A)** Increasing the buffer size from **one** to **three** stimulation sequence lengths (1.05–3.15 s) led to an increase in mean accuracy from 86.76 to 99.21%. **(B)** Of course, the latency of the system depends on the signal buffer length. However, small buffer might reduce the quality of the extracted features. Hence, the latency presented here shows the time that is necessary to achieve classification accuracy above 80%. Increasing the signal buffer size increased the latency from 2.59 to 3.65 s.

Figure 4A shows the mean on-line accuracy for three buffer lengths (1.05, 2.10, and 3.15 s) after 20 trials. The shortest window length resulted in the lowest accuracy, but fastest reaction of the BCI system. The classification accuracies for growing buffer length were 86.76, 92.96, and 99.21%, respectively. Figure 4B illustrates the BCI latency that was necessary to reach 80% classification accuracy. Specifically, this latency consisted of the moving average window length (1.0 s), the signal buffer length and the time that was necessary for the user to locate and focus on the target and then produce clear ECoG activity. The exact latencies were 2.59, 3.14, and 3.65 s for a buffer length of 1.05, 2.10, and 3.15 s, respectively.

### Evaluation of the zero class

**Figure 5:**
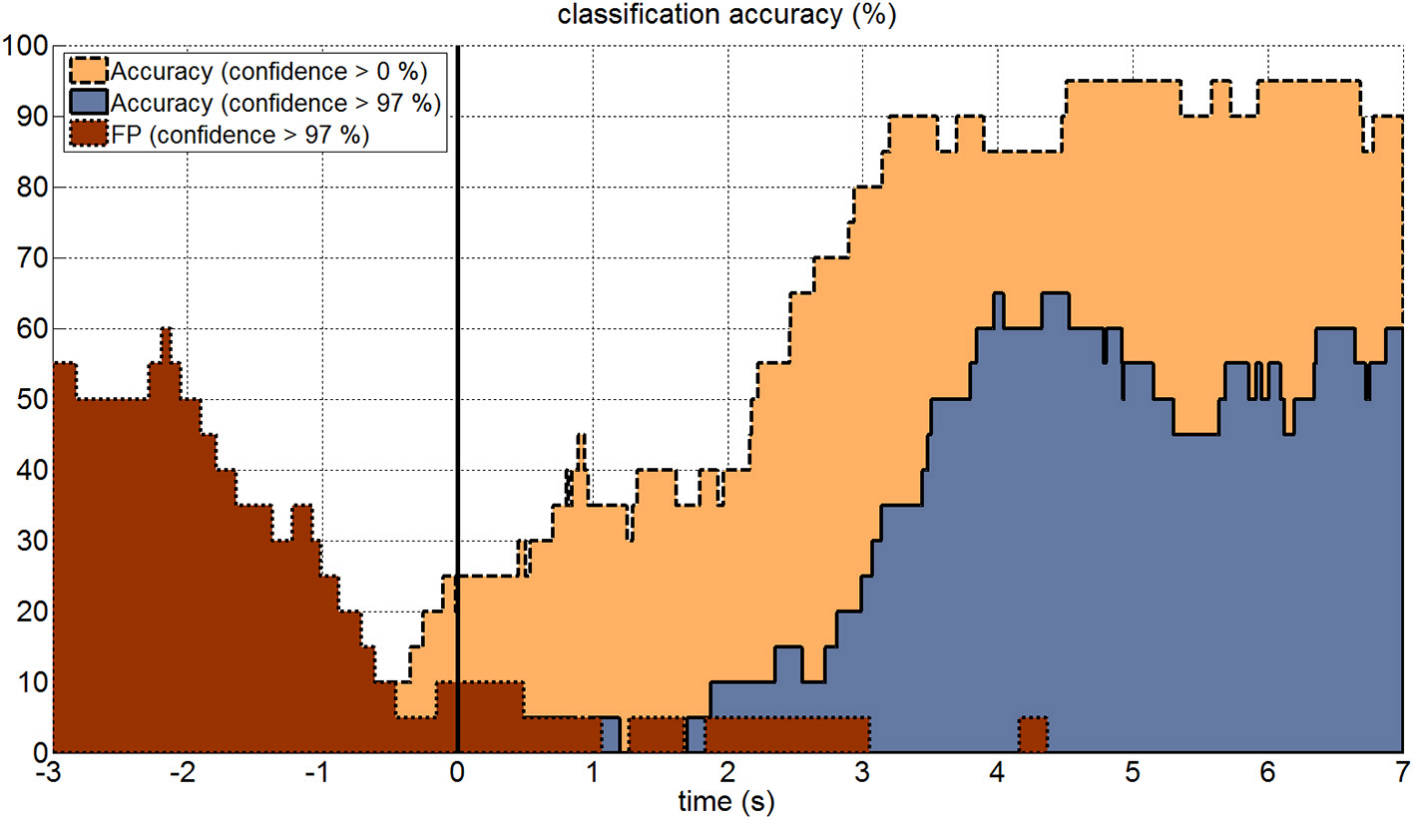
**Online accuracy and zero class**. The figure shows the online classification accuracy for a 10 s long trial. The black vertical line indicates the onset of the visual stimulation for each trial. Classification was performed using a 2.10 s signal buffer and a confidence interval for classification of 0–100% (yellow area with dashed line) and 97–100% (blue area with solid line). The false positive rate (red area with dotted line) was very high before the stimulation onset, because of the buffer overlap from previous trials. Once the signal buffer did not contain any c-VEPs anymore, the false positive rate dropped down to around 5% and then to 0%, when the signal buffer was again filled with c-VEPs.

Figure 5 shows as an example the effect of the zero class for a signal buffer window length of 2.10 s. The highest classification accuracy could be reached when no zero class was applied. However, if the subject was not attending to any target, the resultant false positive (FP) rate was very high and showed random target selection. If the threshold for the zero class was set to a confidence level of 97%, then the classification accuracy was about 30–40% lower, but also FP rate dropped down to less than 5% if the subject was not attending to one of the targets (second 0.0 to 3.0 in Figure 5). Once the subject was focusing a target and the signal buffer contained the corresponding c-VEPs, the FP rate even dropped down to 0%, which means that only reliable features were accepted for classification. The period from second −3.0 to 0.0 represented a period, in which the subject was intended to stop the selection of the previous target. From second −3.0 to −2.0 the FP rate rose up to 50–60%. This period was characterized by high feature values from the last classification leading to an FP rate similar to the classification accuracy with enabled zero class. After another 1.5 s the signal buffer did not contain any target related VEPs anymore.

### Unsupervised online experiment

During the free run, the subject performed 17 unsupervised selections. She reported that the feedback of the selected targets was correct throughout the whole run.

## Discussion

This work successfully showed that a continuous control signal can be extracted from ECoG data with c-VEPs. The c-VEP approach has an advantage over SSVEPs: there is no single frequency that flashes. Instead, a certain code is embedded, and therefore the risk of inducing seizures is reduced, which is especially important for subjects with increased photosensitivity. SSVEP studies showed that the area of the flashing item, the intensity and the frequency are important parameters for improving the performance. But increasing area and intensity are especially problematic for these persons. SSVEP BCIs work best in a frequency range between about 8–25 Hz, which is a sensitive range for inducing seizures. The c-VEPs contain a mixture of frequencies with lower intensity, comparable to watching TV, and are therefore better suited for these persons.

A big problem of ECoG BCI studies is the rapid selection of appropriate channels coding the necessary information. This can either be done offline with channel selection algorithms like distinction sensitive learning vector quantization (DSLVQ) (Pregenzer and Pfurtscheller, 1995) or by observing reactive frequency spectra or similar parameters. But all these procedures require manual optimization and are time consuming. In the presented work, a spatial filter was automatically obtained using the calibration data during the offline analyses. The result is a spatial filter that selects the most important channels according to their importance for the discrimination task. This is comparable to common spatial patterns (CSP) that are used for motor imagery based BCI system to automatically weight the channels (Guger et al., 2000). For this patient, one of the electrodes (channel 95) mostly contributed to the classification task, two more channels showed medium weights for the spatial filter (channel number 27 and 96 ordered by priority). This is similar to CSP, where the weights of the electrodes are also ordered according to the eigenvalues obtained with the algorithm (Müller-Gerking et al., 1999). Interestingly, electrodes 95 and 96 were neighbors located at occipital base (green/blue channel in Figure 1), but electrode 27 was located at lateral frontal lobe (blue channel in Figure 1). However, as the amplitude of the VEPs of the electrodes 95 and 96 were much higher than for electrode 27, the contribution of electrode 27 to the feature values was very small.

The spatial filter also allows easy system configuration updating with new calibration data. Previous publications with EEG data showed that subjects were trained multiple times to increase the classification accuracy (Guger et al., 2012). In an SSVEP group study with EEG recordings from 53 subjects, the classification accuracy could be increased from a grand average accuracy of 87.9–95.5% after only 20 min of training (Guger et al., 2012). In the current study the used c-VEP algorithm showed a high mean classification accuracy of 99.21% that was achieved with only 200 s of calibration data and after only a single training run lasting less than 4 min in total.

In comparison, the group study of Guger et al. (2012) showed that 27 out of 53 subjects achieved perfect accuracy of 100% and only seven subjects achieved less than 90% accuracy. Another study with a c-VEP BCI including 11 subjects reported a grand average classification accuracy of 98.18% with nobody below 90% accuracy (Kapeller et al., 2013). This study showed that a BCI using c-VEPs works even for subjects who could not use an SSVEP BCI. The c-VEP BCI using ECoG showed similar results compared to the EEG study. This might be an indicator that the SNR of the recorded VEPs was similar in both setups. However, it is important to note that the ECoG setup is much more robust against eye blinks or EMG artifacts. Moreover, in this study the signals were recorded from the right hemisphere only and it is unclear if the electrode positions were optimal with respect to the amplitude of the VEPs.

Notably, the mean classification accuracy from second 5 to 7 is 99.21%, which shows that the subject could continue attending to a certain target for a longer time window. This is important for generating a continuous control signal, unlike discrete selection BCI systems such as a typical P300 BCI. Sutter tested an approach similar to the present system with ECoG data in 1992. He used also c-VEPs with 64 icons on a screen for a spelling system. The study showed that 64 targets could be successfully selected by the ALS patient, who reached about 10–12 words a minute with this setup. Nowadays, the P300 is used for most BCI spellers because these BCI systems support a very high number of targets and can work even better if more targets are used (Allison and Pineda, 2003; Sellers et al., 2006; Guger et al., 2009; Brunner et al., 2011). The SSVEP and c-VEP principles are limited by the number of different targets that can be generated. While the screen refresh rate is a limiting factor for an SSVEP BCI, the sequence length defines the number of possible targets in a c-VEP BCI.

Although, the c-VEP principle allows fast switching from one target to another target, the latency of at least 1.05 s requires anticipation of the user, e.g., while steering an avatar or a robotic system in real-time. Previous studies using ECoG showed very short latencies during 3D cursor control (Wang et al., 2013) or movement of a prosthetic arm (Yanagisawa et al., 2012). In these studies the subjects either performed real or attempted movements in order to detect changes in power of different frequency bands, especially in the frequency band >70 Hz. However, such a BCI system that is independent from motor execution usually requires a much longer training period than the calibration time presented in this work. While using the c-VEP BCI, the subject learned very easily and quickly how to control the system. She was able perform a calibration run, a testing run and a free run within 30 min.

In combination with a goal-oriented BCI system, this allows for full control of humanoid robotic systems. For such applications, it is important to embed the BCI controls into a control display that is either a computer screen or a head-mounted display. Such a humanoid robotic system might have also cameras with 2D or 3D vision embedded for online transmission of a video stream to the BCI feedback monitor, so that the BCI user is also embedded in its avatar for optimizing the control behavior (Gergondet et al., 2012). In this case a big advantage is the zero class property, because it ensures that the humanoid robotic system does not make movements if the user is not attending to one of the controls.

We plan to integrate and test different target shapes and patterns and increase the number of targets. Since the number of targets for the c-VEP configuration is only limited by the sequence length and the minimum phase shift between the sequences, the system can be optimized in terms of number of decisions and reaction time.

### Conflict of interest statement

The authors declare that the research was conducted in the absence of any commercial or financial relationships that could be construed as a potential conflict of interest.
